# A Natural Deep Eutectic Solvent Formulated to Stabilize β-Lactam Antibiotics

**DOI:** 10.1038/s41598-018-33148-w

**Published:** 2018-10-08

**Authors:** Belén Olivares, Fabián Martínez, Lina Rivas, Cristian Calderón, José M. Munita, Paola R. Campodonico

**Affiliations:** 1 Centro de Química Médica, Facultad de Medicina, Clínica Alemana Universidad del Desarrollo, Santiago, Chile; 2 Programa de Postgrado en Biología, Universidad Nacional de Cuyo, Mendoza, Argentina; 3 Programa de Genómica y Resistencia Microbiana, Facultad de Medicina, Clínica Alemana Universidad del Desarrollo, Santiago, Chile; 4 Millennium Nucleus on Interdisciplinary Approach to Antimicrobial Resistance, Santiago, Chile

## Abstract

β-lactam antibiotics, such as penicillin share a very unstable chemical structure. In water-based solutions, such as those used for clinical applications, the β-lactam ring is readily opened due to a nucleophilic or electrophilic attack, leading to the loss of antimicrobial activity. Since the achievement and maintenance of optimum therapeutic levels of β-lactam antibiotics is critical for the resolution of many infectious clinical situations, and to avoid antibiotic resistance generation, the design of new non-aqueous dosage forms is urgent. Recently, natural deep eutectic solvents (NADES) have emerged as alternative non-toxic and non-aqueous solvents for different biomedical applications. In this work, we formulated and characterized a NADES composed by betaine and urea (BU). Using this solvent, we evaluated the stability of clavulanic acid (CLV) and imipenem (IMP) and characterized their antimicrobial activities calculating the minimal inhibitory concentration. Characterization of BU solvent by infrared spectroscopy (IR) and nuclear magnetic resonance spectroscopy (NMR) indicated that the obtained solvent has a microstructure mainly based on hydrogen bonding interactions and water addition strongly affects its dynamic. The stability of β-lactam antibiotic IMP and CLV using this solvent was increased by 7 fold and 2.5 fold respectively compared to water when analysed seven days after being dissolved. Microbiological assays showed that antibacterial activity at day seven was significantly decreased for both CLV and IMP when dissolved in water, while no change in their antibacterial properties was observed when antibiotics were dissolved in BU. The increased stability of IMP and CLV in BU may be related to the inert behaviour of the solvent and the higher dynamic restriction that helps antibiotics to maintain a more stable conformation. These data suggest the potential use of BU as a solvent to prevent degradation of β-lactam antibiotics.

## Introduction

β-lactam antibiotics are recognized as one of the most powerful drugs in human history and for many advantageous clinical reasons, such as low toxicity, they remain the most widely used class of antibiotics^1^.

Bacterial killing for β-lactam antibiotics is related to the length of time that bacteria are exposed to a concentration of antibiotic that exceeds the minimum inhibitory concentration (MIC). Moreover, maintaining antibiotic concentrations over MIC is important not only for clinical therapeutic reasons, but also to avoid the generation of antibiotic resistance^2^. Therefore, therapeutic strategies tending to achieve that condition are desirable, for example, continuous infusion has been proposed over intermittent bolus administration^3^. For the same purpose, matrix-type extended-release technology occupies the major market share of sustained-release antibiotic products. Several materials have been developed in the past few decades to obtain matrices that allow the optimum delivery of antibiotics^4^. But for all administration routes, the achievement of an extended release goal of β-lactam antibiotics is limited by the low physicochemical stability of these molecules.

In their chemical structure, β-lactam antibiotics share a very unstable and reactive four-membered ring^5,6^. In aqueous solutions, such as many of those used for clinical administrations, the β-lactam ring is readily opened due to a nucleophilic or electrophilic attack, leading to antibiotic degradation, loss of antimicrobial activity and increased risk of therapeutic failure^7,8^.

Quality specifications for pharmaceutical products generally establish that concentrations of the active antibiotic molecules should be in the 90–110% range of the products label^9^. This requirement is challenging for many extemporaneous aqueous preparations. It was reported that amoxicillin-clavulanic acid syrups maintain its active ingredient stability for only 48 hours when stored at room temperature^10^ and must be strictly refrigerated to assure in-use quality. Also many parenteral dosage forms of β-lactam antibiotics, once reconstituted and solubilized in aqueous solvents have 4 to 8 hours of stability at room temperature^11–13^. Since to achieve and maintain an optimum therapeutic level of β-lactam antibiotics is critical in many infectious clinical situations, there is an imperial need to design new dosage forms by using solvents able to dissolve and maintain stability of the antibiotic active ingredients.

The instability of β-lactam antibiotics in aqueous solution has been known since the time of their discovery^14^. Although there are many physicochemical strategies that tend to increase their stability, like addition of buffer salts to keep them in the pH range of maximum stability, or cation complexings excipients that could prevent them from acting as degradation catalysers. Nowadays there is no substance able to significantly prevent degradation of β-lactam molecules, allowing them being in solution for long periods.

Recently, natural deep eutectic solvents (NADES) have emerged as alternative non-toxic solvents for different biomedical applications, due to their natural components and projected biocompatibility. They are prepared with a combination of two or more solid substances, which, when mixed in appropriate proportions, give rise to a mixture with a lower melting point compared to the melting point of the individual constituents. Thus resulting in a new liquid chemical specie^15^.

For some authors NADES represents a greener generation of the ionic liquids (ILs) because they have a pseudo ionic fluid state and as ILs, they give the possibility of designing matrixes with desired physicochemical properties using different constituents and proportions^16^. In that field there are many studies evaluating new pharmaceutical applications for NADES^17–20^.

The goal of the present work was to formulate, prepare and characterize a NADES able to stabilize two unstable β-lactam molecules: imipenem (IMP) and clavulanic acid (CLV) considered as the most unstable molecules used in the antibiotic parenteral and oral therapy, respectively^21,22^.

## Materials and Methods

### Drugs and Reagents

The antibiotic drugs under study were clavulanic acid (CLV) (gift of SAVAL SA Laboratory, Chile) and Imipenem monohydrate (IMP) (USP Cat. N 1337809, Sigma-Aldrich). For NADES preparation betaine monohydrate 98% (Santa Cruz Biotechnology) and urea 98% (Merck) were used. Ultrapure water was obtained with a purification system (Simplicity^®^ Millipore). For microbiological studies, Amoxicillin trihydrate (AMX) (Sigma Aldrich), Mueller Hinton II and Muller Hinton Broth (cation adjust) (Becton Dickinson) were used.

### NADES preparation

The NADES was prepared with betaine and urea by heating and mixing as previously described^23^. Betaine and urea were mixed in a 1:1.5 molar on water bath at 60 °C. The clear liquid obtained was left resting while its appearance was monitored for fifteen days until its characterization.

### BU characterization

The FT-IR spectra of the samples were registered in the 4000–400 cm^−1^ region using a Bruker IFS 66 v instrument (Bruker, Coventry, UK). Nuclear magnetic resonance spectroscopy (NMR) measurements were recorded on a Bruker Avance 400 spectrometer (Bellerica, MA, USA) operating at 400.13 MHz (^1^H) and 100.62 MHz (^13^C) at 25 °C using D_2_O as solvent; 32 and 10.000 scans were taken for ^1^H and ^13^C, respectively.

For NMR recording, a non-diluted sample of BU was placed directly in capillary tube. Urea and betaine 4%w/w D_2_O solutions were prepared and separately measured. Water signal was used as an internal standard for spectral calibration.

### CLV and IMP stability study

Solutions of the drugs were prepared in distilled water and in the BU by mixing the solvent and solutes in vortex for two hours and then were kept at 25 °C +/− 2 °C for seven days in a water bath circulation system (PolyScience, USA). Samples were taken at 0, 1 and seven days and stored at −80 °C until analysis.

### Chemical stability evaluation

High Performance Liquid Chromatography (HPLC) analysis was performed, for each molecule under study, based on previous reported methods^24,25^. CLV and IMP were measured using a VWR Hitachi LaChromElite equipped with column oven, auto sampler, and UV-DAD detector. Samples were diluted with distilled water before analysis. Reversed phase runs for CLV analysis comprised a mobile phase of 85% 20 mM potassium bi-acid phosphate adjusted to pH 7 and 15% methanol (Merck), at a flow rate of 0.7 ml/min with a Kromasil® C18 3.5 µm 150 mm × 4.6 mm column (AkzoNovel). For IMP analysis, the chromatographic conditions consider the mobile phase described earlier with 5% methanol adjusted to pH 5 with ortho-phosphoric acid. A flow rate of 0.8 ml/min on a C18 5 µm 150 × 4.6 mm ACE Generix® column was used. Stability studies were made considering the areas of the chromatographic peaks, due to its direct relation with the quantity of the intact drug. Final measures considered the following formula:

$$ \% \,{remaining}\,{drug}=\frac{Are{a}_{t}}{Are{a}_{0}}\times 100$$

where Area_t_ is the area of the chromatographic signal measured, which is directly related to the intact drug content in each sample for a specific sampling time. Area_0_ is the signal measured initially in each sample just after its dissolution in the stability study.

### Solubility evaluation

Solubility studies were performed by mixing the β-lactam antibiotics with BU or with water. Briefly, an excess of IMP and CLV were added to 5 ml of solvent and stirred at 450 rpm using an orbital mixer (mLab scientific HCM 100-pro) for 24 hours at 25 °C. Finally samples were filtered with a 0.2 µm nylon membrane and diluted to perform HPLC concentration measurements.

### Antimicrobial *in vitro* activity determination

Antimicrobial activity of CLV and IMP were assessed at 0 and seven days after being dissolved in BU or water against *Escherichia coli* ATCC-35218 and *Pseudomonas aeruginosa* ATCC-27853 strains respectively by two sensitivity methods: Broth microdilution test (BMD), and Disk diffusion (DD). For the BMD test, antimicrobial activity was evaluated by calculating the minimal inhibitory concentration (MIC). Strains were cultured on agar Mueller Hinton Broth. 0.5 McFarland inoculum was seeded with 50 µl of the liquid medium and 50 µl of the drug solutions for 18 hours. Drug solutions were prepared to attain the following final concentrations for AMX/CLV: 128/64, 64/32, 32/16, 16/8, 8/4 4/2, 2/1, 1/0.5 and 0.5/0.25 µg/ml respectively. The same concentrations of AMX without CLV were prepared to verify β-lactamase phenotype. IMP solutions were prepared to attain the following final concentrations: 32, 16, 8, 4, 2, 1, 0.5, 0.25 and 0.125 µg/ml.

The DD test antimicrobial activity was evaluated measuring the diameter of the inhibition zone generated by the drugs dissolved in water and in BU loaded on cellulose disks. Drug solutions were freshly prepared in the following concentrations: 2000, 1000 and 1000 µg/ml of AMX, CLV and IMP respectively. Then, 10 µl of the antibiotic solutions were loaded on the corresponding blank disks under sterile conditions, resulting in AMX-CLV 20/10 µg; CLV 10 µg and AMX 20 µg disks. Disks were then incubated for 24 hours on an agar Mueller Hinton II plate seeded with 0.5 McFarland inoculum of *Escherichia coli*. For IMP evaluation, disks containing 10 µg of the drug were incubated for 24 hours on an agar Mueller Hinton II plate seeded with 0.5 McFarland inoculum of *Pseudomonas aeruginosa*.

### Statistical analysis

Data were presented as mean ± standard error of the mean (SEM). Multiple group comparisons were performed by analysis of variance (ANOVA) followed by Bonferroni *post hoc test*, while comparisons between two experimental groups were performed by Student’s t test. p < 0.05 was considered statistically significant. All experiments were carried out in triplicate.

## Results and Discussion

### NADES selection and preparation

There are two aspects driving the stability results: the potential harmful of the solvent molecules and the sensitivity of certain conformations of the B lactam molecules that make them more sensible). The NADES design was based on the following hypothesis: the eutectic mixture represents an inert network where β lactam molecules could adopt a more stable, (not sensible to solvent attack) and restricted conformation (not changing) than the adopted when water is used as solvent.

To formulate a NADES, with the mentioned characteristics natural substances and proportions previously reported as able to generate eutectics^26^ were selected. The use of alcohols or carbohydrates as part of this NADES were avoided because of the confirmed hydrolytic nature towards β-lactam ring^27^. We specifically focused on Baskakov *et al*., Kumar *et al*. and Zeng *et al*. research^28–30^, who have described the use of betaine-urea mixtures for stabilization of protein structure. Thus, those substances were selected as the starting point to formulate the solvent and evaluate our hypothesis.

Many betaine and urea molar ratios have been reported with the capability of forming liquids^31^. Nevertheless, the ratio containing the minimum urea content that remained stable at 25 °C was used, based on the pre-assumption that the more urea present, the more adverse could be the solvent, because of its nucleophilic nature^28,32^. Accordingly, 1 to 1.5 betaine-urea (BU) molar ratio was selected.

Heating was applied as the preparation method because it offers an easy and feasible way to prepare the eutectic mixture and to control the final water content^26^. Therefore, betaine and urea were mixed while continuously heating at 60 °C for two hours, until a clear liquid was formed.

It has been described that not only the molar ratio of the main constituent of the eutectic formula is important, but, also, the water content is a key factor in the physicochemical properties of the final formulation. It has been reported that water can modify the polarizability and basicity of the H-bond acceptor and that H-bond is one of the main tuneable interactions in the solvent^33^. Thus, 0, 0.3, 2, 5, 10, and 15% w/w of water were added to different aliquots and mixed. The resultant mixtures were kept at room temperature for fifteen days for macroscopically stability observation.

After the observation period it was found that the aliquot without water addition and with 0.3% w/w of water presented crystal precipitates. All the other mixtures maintained their liquid and homogeneous states with the more water proportion the less viscosity (macroscopically evaluated). That result indicates that water addition is necessary to maintain diffusion of the betaine and urea molecules that allow the molecular interactions needed to maintain the stability of the matrix. Water apparently acts as plasticizer in the matrix as has been observed in amorphous semi-solid materials^34^.

### BU Characterization

#### NMR analysis

**Figure 1:**
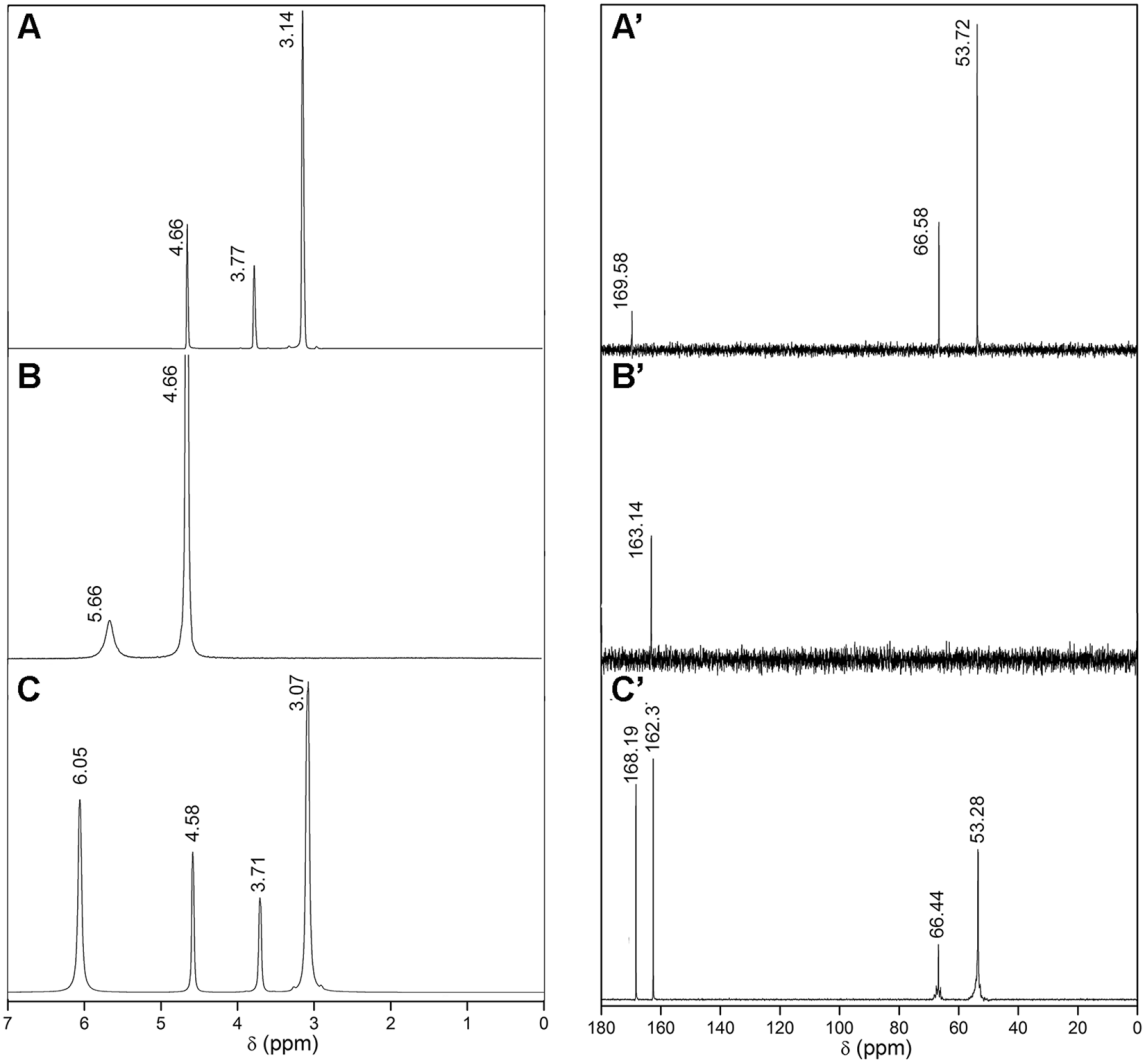
^1^H NMR spectrums of betaine (**A**), urea (**B**) and BU (**C**); and ^13^C NMR spectrums of betaine (A’), urea (B’) and BU (C’) respectively. Spectra were recorded at 27 °C (300 K).

To elucidate the identity and molecular interactions involved, ^1^H and ^13^C NMR analysis was performed on BU prepared with 2% w/w of added water. This technique represents a selective and non-invasive approach commonly applied for NADES characterization^35,36^. Figure 1A–C depict the ^1^H and Fig. 1A’–C’ the ^13^C NMR spectrums of betaine, urea and BU respectively. The ^1^H NMR spectrum of betaine (Fig. 1A) showed the characteristic methylene protons signal at 3.77 ppm and methyl groups at 3.14 ppm, while the ^13^C NMR spectrum (Fig. 1A’) showed three signals at 169.58, 66.58 and 53.72 ppm assigned to the carbons present on carbonyl, methylene and methyl groups respectively. For the urea (Fig. 1B) the ^1^H NMR spectrum showed one broad signal assignable to –NH_2_ protons at 5.66 ppm. The broadening of the signal is related to the quadrupolar effect of the nitrogen atom, while the ^13^C NMR spectrum (Fig. 1B’) shows only one signal assigned to the carbonyl carbon.

The BU ^1^H spectrum, showed four signals (Fig. 1C). The signal at 4.58 ppm assigned to the water molecules in the sample are, in comparison with betaine and urea individual ^1^H spectrums (Fig. 1A,B), shifted to a higher field due to the change in the magnetic environment present in BU. In addition, the other three signals in BU show a broadening effect and downfield shift compared with the starting materials.

The observed chemical shifts also have been previously described in other NADES preparations^30,37^. The evidence of hydrogen bonding is supported by the fact that betaine in BU shows a poorer electron density over the nitrogen atom; meanwhile, urea has a higher electron density over its oxygen atom. This difference in charges distribution enables the formation of energetically favourable pairings areas, which could support the existence of strong hydrogen bonding between betaine and urea in the liquid.

The differences in the spectrum patterns between BU and the individual components are due to the different shielding of nuclei by electrons, showing at least two forms of molecular ordering structures in BU; one of them possibly attributed to the high proportion of intermolecular interactions (mainly hydrogen bonding). These results are in agreement with those previously reported by Kumar *et al*., who have described the same interaction type by molecular dynamics studies^38^.

#### FT-IR analysis

**Figure 2:**
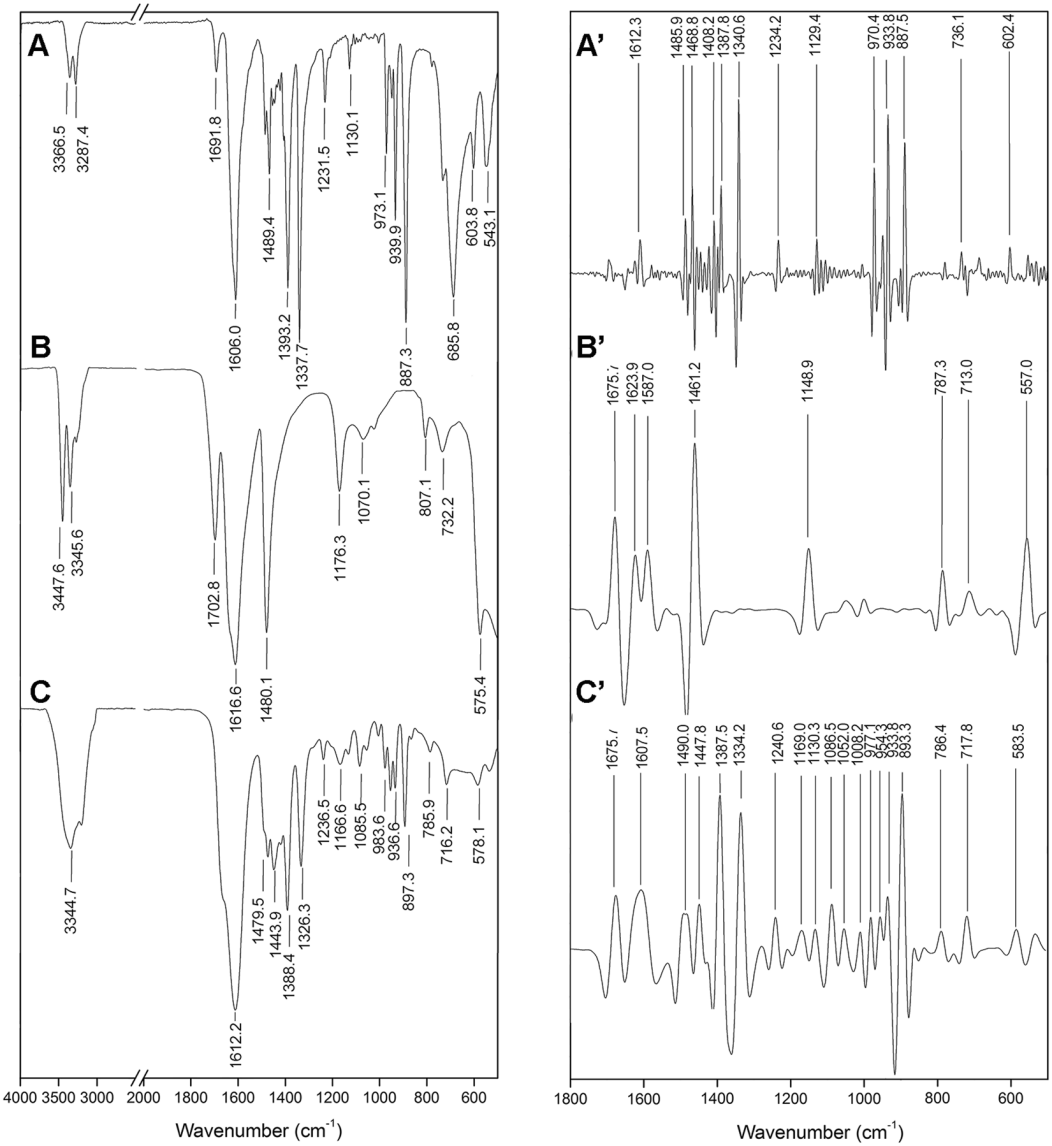
Vibrational characterization of betaine (**A**) urea (**B**) and BU (**C**) through FT-IR spectroscopy and second derivative spectrums, respectively (A’, B’ and C’).

Figure 2 depicts the FT-IR spectrum of betaine (A), urea (B), and BU (C) together with their more resolved second derivative in 2000–500 cm^−1^ (A’, B’, C’ respectively).

The FT-IR spectrum of betaine (Fig. 2A) presented the characteristic bands of N-C asymmetric and symmetric stretching at 3336.5 and 3287.4 cm^−1^, respectively. Furthermore, some important bands of amine groups (N-C stretching vibrations) can be observed as more resolved in the second derivative of the FT-IR spectra (Fig. 2A’). At 1606.0 and 1489.4 cm^−1^ the two characteristics vibrations attributed to asymmetric and symmetric stretching bands of carboxylate groups could be observed. The FT-IR spectrum of urea (Fig. 2B) depicted bands at 3447.6 and 3345.6 cm^−1^ assigned to N-H asymmetric and symmetric stretching. In comparison with betaine, the major wave numbers for this vibrational mode are related to the presence of the carboxyl group that affords a vibration with higher energy. The band at 1702.8 cm^−1^ is assigned to the carbonyl group stretching. The spectra showed two characteristics bands assigned to amide groups (1616.6 and 1480.1 cm^−1^) for amide I and II. Due to the simplicity of the urea molecule, the second derivative of FT-IR spectra (Fig. 2B’) did not show additional information.

**Figure 3:**
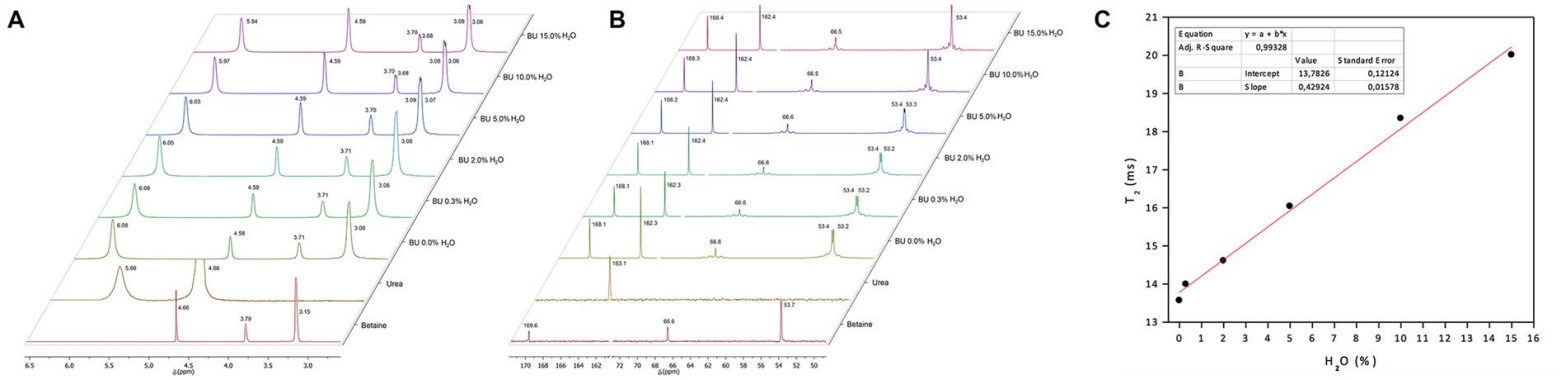
^1^H NMR (**A**), ^13^C NMR (**B**) characterization of betaine, urea and BU prepared with different water proportion. Part C shows transversal time (T_2_) evolution of urea proton with the variation of water content in BU. Data were obtained from three independent determinations.

The Fig. 2C shows the FT-IR spectrum of BU. It was similar to the corresponding betaine and urea spectrums; nevertheless, certain bands were broader and shifted to lower wave numbers. The N-H asymmetric and symmetric stretching bands appeared as a broad band at 3347 cm^−1^. This broad band is the result of two phenomena: the overlapping of asymmetric and symmetric N-C and N-H stretching for betaine and urea respectively, and the hydrogen bonding formation. The latter gave rise to two characteristics effects: the lowering of the vibration frequency, and the broadening of the bands. Another band that showed this effect was the C-N deformation vibration at 1444.9 cm^−1^. At 1612.2 cm^−1^ a strong and broad band assigned to the carbonyl group appeared. In the second derivative of FT-IR spectra (Fig. 3C’) this band appeared resolved in two bands: the carbonyl stretching (1675.7 cm^−1^) and the amide stretching (1607.5 cm^−1^). These bands showed the lowering effect due to the hydrogen bonding.

The evidence through NMR and FT-IR analysis, of the hydrogen bonding in the BU prepared with 2% w/w of water indicates that the solvent has an interaction feature between betaine and urea that could promote the protective characteristics previously reported^29^.

### Water effect on the BU matrix

#### Water modifies betaine - urea interactions

Under the preparation procedure followed to obtain BU, at least 2%w/w of water addition is needed for maintaining a stable and homogenous liquid. Nevertheless, it has been described that water addition results in less viscosity^39^, which could impact in some desirable properties, including good material transfer. This property is an essential requirement for mixing operations and in drug release processes to give optimum bioavailability characteristics of the therapeutic drug^40,41^.

On the other hand, previous observations showed that betaine-urea interaction is concentration dependent^30^, and the hydrogen bonding between them is weakened by high water content^35^. Thus, adjusting the water proportion without losing the intermolecular interactions is a critical task.

To analyse the effect of water in BU interactions, NMR studies were performed in several BU preparations containing up to 15% w/w water addition. Figure 3 depicts the ^1^H and ^13^C NMR spectrums of BU samples prepared, all obtained using the same instrumental parameters (see methods section).

As shown in ^1^H and ^13^C NMR spectra described in Fig. 1, the proton signals of BU present chemical shifts in comparison with urea and betaine individual molecules (Fig. 3A): the urea proton showed an important chemical shift to low field, but betaine protons were slightly shifted to high field.

The observed shifts in all spectra confirmed the presence of hydrogen bonding interaction between betaine and urea. Nevertheless, urea signals become narrow as the water content increases in the BU preparation (Fig. 3A around 6.0–5.6 ppm). This effect can be explained due to the disruption of hydrogen bonding between urea and betaine, leaving only one form of interaction present in the matrix.

It can also be observed that the increase in water content induces changes in the signals shapes for betaine protons (Fig. 3A around 3.8–3.06 ppm). At low water concentration, less than 2% w/w, the signals showed a Gaussian overlapping shape, meanwhile higher water proportions split the signal. This result suggests a betaine participation in hydrogen bonding with urea and water that, at the same time, leads to at least two magnetically different environments for the nucleus.

The ^13^C NMR spectra for BU (Fig. 3B) showed slight shifts toward higher field with respect to betaine and urea molecules. This observation is related to the indirect effect of hydrogen bonding over the carbons electron density. However, betaine signals showed particular shapes with the increase of water content (Fig. 3B around 53,7–53,4). At lower water content the methyl carbons appeared as a split signal with 0.2 ppm of gap. At higher water content the signal became only one. Moreover, at low intensity, several new signals indicating the existence of new magnetic environments for betaine molecule probably caused by an augmented interaction with water molecules can be observed.

Both ^1^H NMR and ^13^C NMR results suggest a disruption or discontinuity of the BU net with increased amount of water molecules inserted in the solvent. These observations are in agreement with the behaviour attributed to water in NADES previously reported using other technical approaches^35^.

#### Water content modifies the dynamic of BU

As described above, BU interactions disruption in high water content samples results in a greater variability in intermolecular interactions and possibly in a higher dynamic behaviour of its molecules. Thus, it seems reasonable to consider that the nucleophilic nature of urea molecules possibly became enhanced in a more disordered or less restricted network. Therefore, we wanted to assess a potential adverse effect of the observed disruption towards the β-Lactam molecules.

The effect of water on the dynamic behaviour of the component molecules in the liquid matrix can be studied by ^1^H NMR relaxometry. The NMR relaxation and line shape of the signals are an indication of the field heterogeneities. In liquids, the relaxation of the magnetic polarization of a system of equivalent nuclei can be described by two different time constants: longitudinal relaxation time (T_1_), and transverse relaxation time (T_2_). The relaxation time (T_1_) describes the flow of energy between the nuclear spin system and the other degrees of freedom of the system known as the lattice. Transverse relaxation time is dependent of direct interactions between the spins of different nuclei without energy transfer to the lattice. Therefore, NMR relaxation is highly sensitive to the dynamics of molecules and has been employed to characterize structured media in which the protic molecules relaxation time strongly depends on the hydrogen bonding interactions^42^.

Bound or motional restricted molecules may exhibit slow reorientation on a more distinguishable scale than free molecules. In this context, we wanted to analyse how free urea molecules are in a BU matrix with different water content. Or in other words, to assess how available urea molecules are in BU with different water proportions.

The NMR results gave the opportunity to choose the formula with less probability of amylolysis of urea towards of β-lactam ring molecules once in it^43^.

Accordingly, NMR relaxometry studies of the urea proton in BU were carried out on the previous prepared water proportion samples (0–15% w/w water). The transversal relaxation time (T_2_) was determined by the total observed width of the signals (ΔU_1/2_)_,_ according to the following equation:

$${\rm{\Delta }}{\nu }_{1/2}=\frac{1}{\pi {T}_{2}}$$

The relationships of longitudinal transversal time (T_2_) of urea with BU water content are shown in Fig. 3C. The NMR relaxometry results showed an increase of transversal time while increasing water content in the eutectic solvent preparation. In both cases, the increase in T_2_ relaxation time with the increase of water concentration is related to the rupture of the hydrogen bonding interaction between the urea and betaine molecules. This phenomenon can only occur if there are specific interactions between the nuclei that result in energy exchange. Due to their immobilized nature in low water content, the urea and betaine are subjected to ^1^H–^1^H dipolar interactions, which make energy transfer very efficient and lead to shorter relaxation time. In summary, the water incorporation during BU preparation could allow urea molecules to become less engaged with betaine, increasing its transversal relaxation time. This observation indicates a similar behaviour of urea as reported in other eutectic mixtures when water content is increased^44^.

### BU increases CLV and IMP stability

Based on the observed results predicting BU with 2%w/w water content as the solvent formulation more closely related with the desired features, we proceeded to evaluate the chemical stability of selected β-lactam molecules in it.

HPLC is the gold standard technique for evaluating stability due to its ability to separate impurities and selectively quantify the molecules of interest^45^. Thus, we developed two HPLC methods to evaluate chemical stability of CLV and IMP in BU based on previously reported methods^13,25,45^.

**Figure 4:**
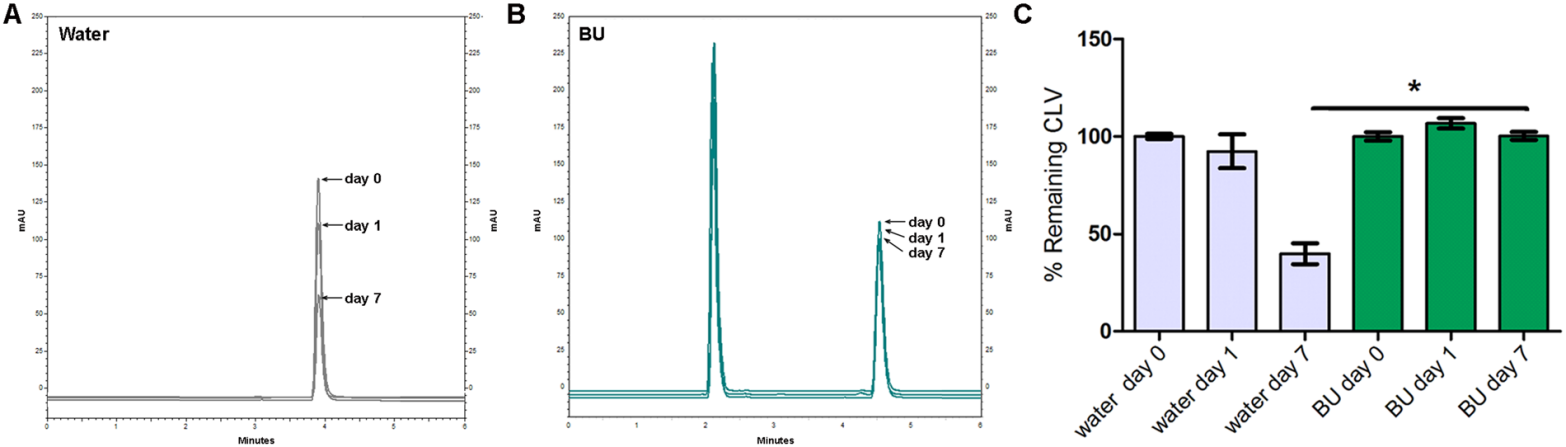
Representative chromatograms obtained of clavulanic acid (CLV) dissolved in water (**A**) and in BU (**B**) of samples of 1 mg/ml after 0, 1 and 7 days. (**C**) Shows chromatogram quantification. Results are expressed as median ± SEM of three experimental replicates.

**Figure 5:**
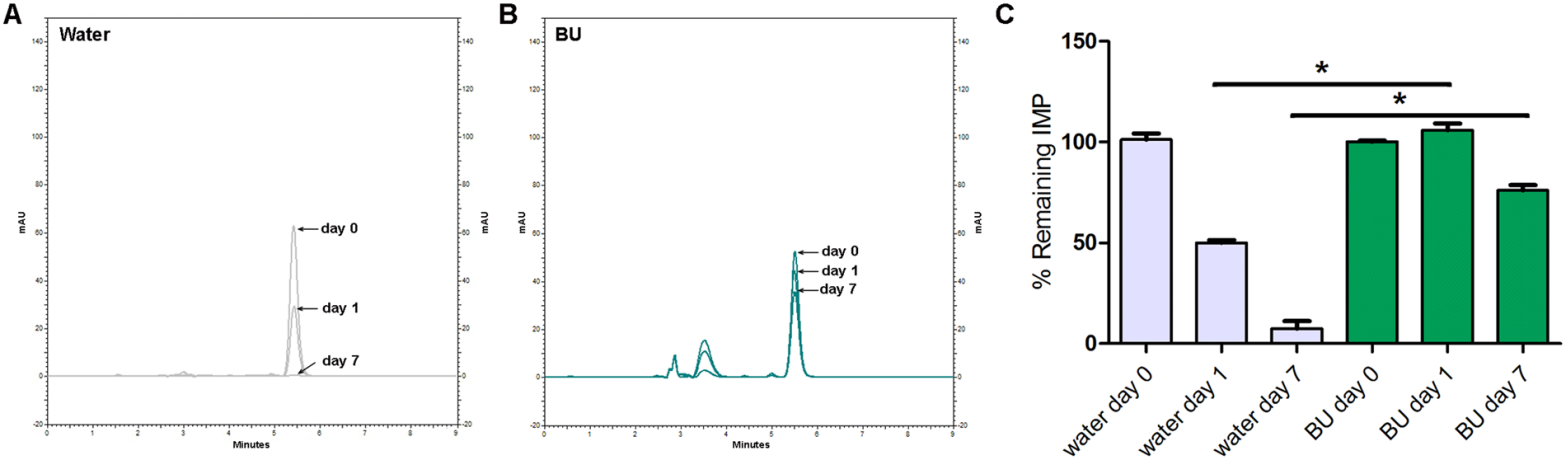
Representative chromatograms obtained of imipenem (IMP) dissolved in water (**A**) and in BU (**B**) of samples of 1 mg/ml after 0, 1 and 7 days. (**C**) Shows chromatogram quantification. Results are expressed as median ± SEM of three experimental replicates.

**Figure 6:**
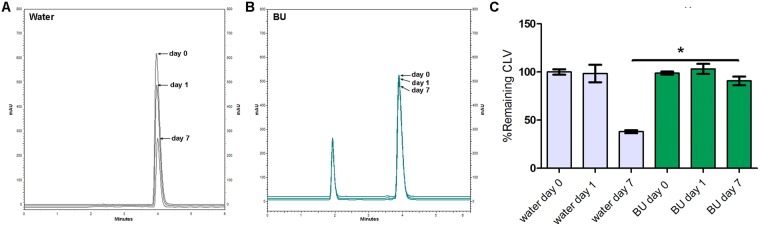
Representative chromatograms obtained of clavulanic acid (CLV) dissolved in water (**A**) and in BU (**B**) of samples of 11 mg/ml after 0, 1 and 7 days. (**C**) Shows chromatogram quantification. Results are expressed as median ± SEM of three experimental replicates.

**Figure 7:**
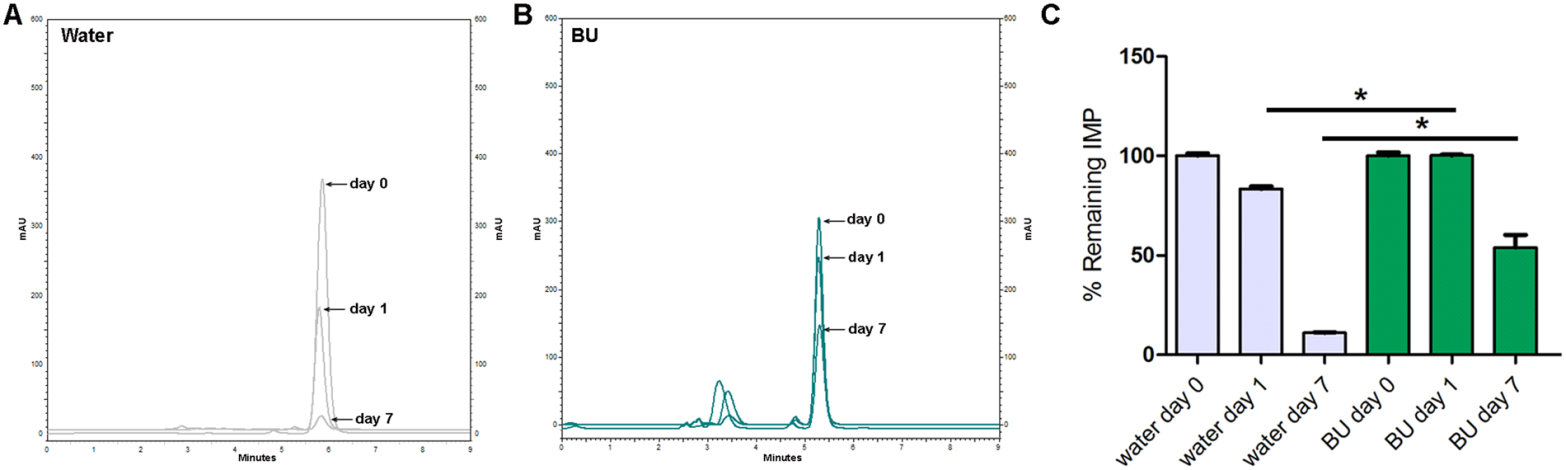
Representative chromatograms obtained of imipenem (IMP) dissolved in water (**A**) and in BU (**B**) of samples of 4 mg/ml after 0, 1 and 7 days. (**C**) Shows chromatogram quantification. Results are expressed as median ± SEM of three experimental replicates.

Figures 4–7 show the chromatographic results obtained after the stability studies of CLV and IMP. Figure 4 depicts overlapping chromatograms obtained from samples of CLV in water (Fig. 4A) and in BU (Fig. 4B) at days 0, 1 and 7. Notice that in Fig. 4B the second peak corresponds to CLV, while the first peak is the BU signal.

CLV in concentration of 1 mg/ml dissolved in BU, maintained stability (quantities of 100.2 ± 1.2% of the initial concentration) after 7 days at 25 °C (Fig. 4B,C), while the remaining CLV quantities after the same time period but dissolved in water was 39.9 ± 5.4% of its initial concentration at day 0 (Fig. 4A,C).

IMP in concentration of 1 mg/ml in BU, maintained quantities of 76.9 ± 3.5% of the initial concentration after 7 days at 25 °C (Fig. 5B,C), while the remaining IMP quantities after the same time period, but dissolved in water, was 7.4 ± 3.8% of its initially measured concentration at day 0 (Fig. 5A,C). For both β-lactam molecules, the BU matrix significantly prevents their degradation.

The same stability study was performed at higher concentrations of the β-lactam antibiotics, in order to evaluate stability in similar concentrations to those used clinically. Thus, CLV and IMP were dissolved in water or BU at 11 mg/ml and 4 mg/ml respectively^46,47^.

When the above-mentioned higher concentrations were studied, similar results were obtained showing that CLV 11 mg/ml in BU after 7 days at 25 °C, maintained 90.7 ± 4.5% of its initial concentration (Fig. 6B,C). While the remaining CLV quantities after the same time period but dissolved in water were 38.0 ± 1.5% of their initial concentration measured at day 0 (Fig. 6A,C).

Figure 7A–C showed similar results for IMP studied in BU and water at 4 mg/ml: 54.0 ± 6.2% of the initial concentration and 11.0 ± 2% respectively.

Another interesting observation was that at day 1, in both concentrations assayed, IMP maintained quantities above 90% of its initial concentrations, showing significant differences from that observed at day 1 in water (Figs 5C and 7C).

The increased stability observed for CLV and IMP when dissolved in BU may be related to the inert behavior of the solvent molecules and the dynamic restriction allowing them to get and maintain a stable conformation. Accordingly, other reports, have revealed a protective effect of betaine towards protein denaturation^48–50^. They have generated evidence of unfavorable betaine interactions with amide carbonyl groups extensively present along the backbone of proteins, which is the same sensible center in the β lactam ring. Similar effect was also reported by Adamczak and co-workers^51^ who recognized betaine as a stabilizing additive of proteins in solution. Interestingly, a synergistic effect between betaine and urea has also been proposed, which further enhances the stabilizing effect of betaine, at the same time that reduces the chaotropic behavior of urea in proteins^52^. Therefore, the data suggest that betaine in BU not only is inert towards CLV and IMP molecules but also plays an important protective role on their reactive centers by an adverse microenvironment.

Additionally, the structure of a NADES is intrinsically different compared to a molecular solvent like water. The former is characterized by structured clusters^53^, with the formation of a tighter network of hydrogen bonding, both in number of interactions and in the polarization degree of the molecules participating in the bondings^33^. Thus, leading to a liquid where the molecules have a restricted movement capacity^54^. The high capability of NADES to form a hydrogen-bonding network had been addressed as an important mechanism for the stabilization of other compounds^55^.

In the present work, the presence of hydrogen bonding network in the BU formulation have been evidenced by FT-IR and NMR analysis. This observation is in complete agreement with a previous study using the same molecules which reported a reduction in diffusion coefficients of the molecules and an increase in the rigidity of the matrix^38^.

Finally, another aspect to consider is that the thiazolidine ring of the penam group in β lactams antibiotics could adopt two conformation (axial or equatorial) that can modulate the stability against nucleophilic attack^56^. The provided rigidity of the BU matrix could give restriction to conformational changes, inducing CLV and IMP to adopt and maintain a more stable conformation, than they could adopt and maintain when dissolved in water.

### CLV and IMP are completely dissolved in BU

An important issue to assess is the solubility of CLV and IMP in BU to elucidate whether the enhanced chemical stability is based on its physical state. It is known that many solid β- lactam drugs have much lower degradation kinetics than those in solution^11^. Therefore, solubility studies were carried out and solubility concentrations of CLV and IMP in BU were determined after 24 hours.

We found similar concentrations of IMP dissolved in BU measured after 24 hours compared to IMP dissolved in water (11325 ± 523 µg/ml in BU vs. 9925 ± 229 µg/ml in water).

In the case of CLV we found less solubility in BU in comparison with water (173618 ± 7067 µg/ml in BU vs. 289447 ± 2947 µg/ml in water). Nevertheless, the solubility concentrations are far above the highest concentrations evaluated during the stability study (approximately 4000 µg/ml of IMP and 11000 µg/ml of CLV). Thus, we confirm that IMP and CLV were completely dissolved in BU. These results indicate that other mechanism of protection different from keeping the molecules in its solid state is occurring.

### CLV and IMP are therapeutically active and available in BU

There are correlations reported between β-lactam ring stability and antibiotic activity describing the therapeutic drug: more instable, the more active^14^. Moreover, it could be considered that BU would give a different conformation from the stereo specificity of the carboxyl groups bound to the carbon of the thiazolidine ring, as well as to the geometry of the fused ring systems described as critical requirement affecting activity^57^. Thus, it is necessary to evaluate whether the antimicrobial activity of the antibiotics is conserved after being dissolved in BU. For that purpose, *in vitro* microbiologic activity tests were performed.

BMD is the gold standard method for quantitatively evaluating antimicrobial sensitivity toward antibiotic drugs and, BMD is based on bacterial growth determinations in culture media containing two folds of antimicrobial drug concentrations. In this method, results are reported as the minimal inhibitory concentration (MIC) of the antimicrobial that inhibit bacterial growth^58^.

Since the focus of this work is on CLV and IMP drugs and not on the sensitivity of the microorganism, we performed the *in vitro* tests following standardized microbiology conditions previously described for clinical laboratory quality controls specified in the Clinical Laboratory Standard Institute (CLSI) compendium^59^. Accordingly, CLV activity was assayed together with amoxicillin (AMX) against *Escherichia coli* ATCC-35218 strain and IMP against *Pseudomonas aeruginosa* ATCC-27853 strain.

Table 1 shows BMD sensitivity test results obtained with freshly prepared (Day 0) drug solutions (BU and water) and with the same solutions but at day 7 after storage at 25 °C.

**Table 1:** Minimal inhibitory concentrations of imipenem and clavulanic acid for *Escherichia coli* ATCC-35218 and *Pseudomonas aeruginosa* ATCC-27853.

Minimum Inhibitory Concentration				
Solvent	IMP		CLV	
	day 0	day 7	day 0	day 7
water	1 µg/ml	≥32 µg/ml	4 µg/ml	8 µg/ml
BU	1 µg/ml	1 µg/ml	4 µg/ml	4 µg/ml
Susceptibility range*	1–4 µg/ml		2–8 µg/ml	

Results are expressed as median of three experimental replicates. The breakpoints ranges established by the Clinical Laboratory Standard Institute (CLSI) are also indicated. *Derived from CLSI specifications.

The MIC for CLV and IMP dissolved in BU and in water at day 0 were 4 µg/ml and 1 µg/ml respectively, thus affording the same susceptibility of bacteria in front of the drugs regardless of the solvent used.

However, activities of CLV and IMP at day 7 were significantly decreased when dissolved in water showing MICs values of 8 µg/ml and 32 µg/ml respectively, while no reduction in their antibacterial properties were observed when dissolved in BU, maintaining the 4 µg/ml and 1 µg/ml MICs values (Table 1).

Considering that the BMD test contemplates two-fold concentrations results, it can be observed that these results are congruent with HPLC chemical quantifications (Figs 4 and 5), confirming that CLV and IMP maintain their therapeutically active structures in BU solutions after 7 days of storage at 25 °C.

It is important to note that during the BMD test procedure, CLV and IMP dissolved in BU and water had to be diluted in the aqueous culture medium, breaking the original BU matrix interactions. Considering that CLV and IMP dissolved in BU could have a different solvation feature and thus different conformation state, it remained an issue of concern to evaluate whether they maintain their therapeutic activity while being dissolved in BU.

To evaluate the antimicrobial activity of CLV and IMP without modifying BU as the primary solvent during the test, disk diffusion (DD) experiments were performed. In this procedure the antibiotic solutions are supported on dry cellulose membrane disks from which they exert action on bacteria inoculated on solid agar plate. Then, after 18 hours of incubation, the disks are surrounded by inhibition zones that are directly related to the antimicrobial activity^60^. Accordingly, fresh BU or water solutions were prepared to perform the test following CLSI specifications.

**Figure 8:**
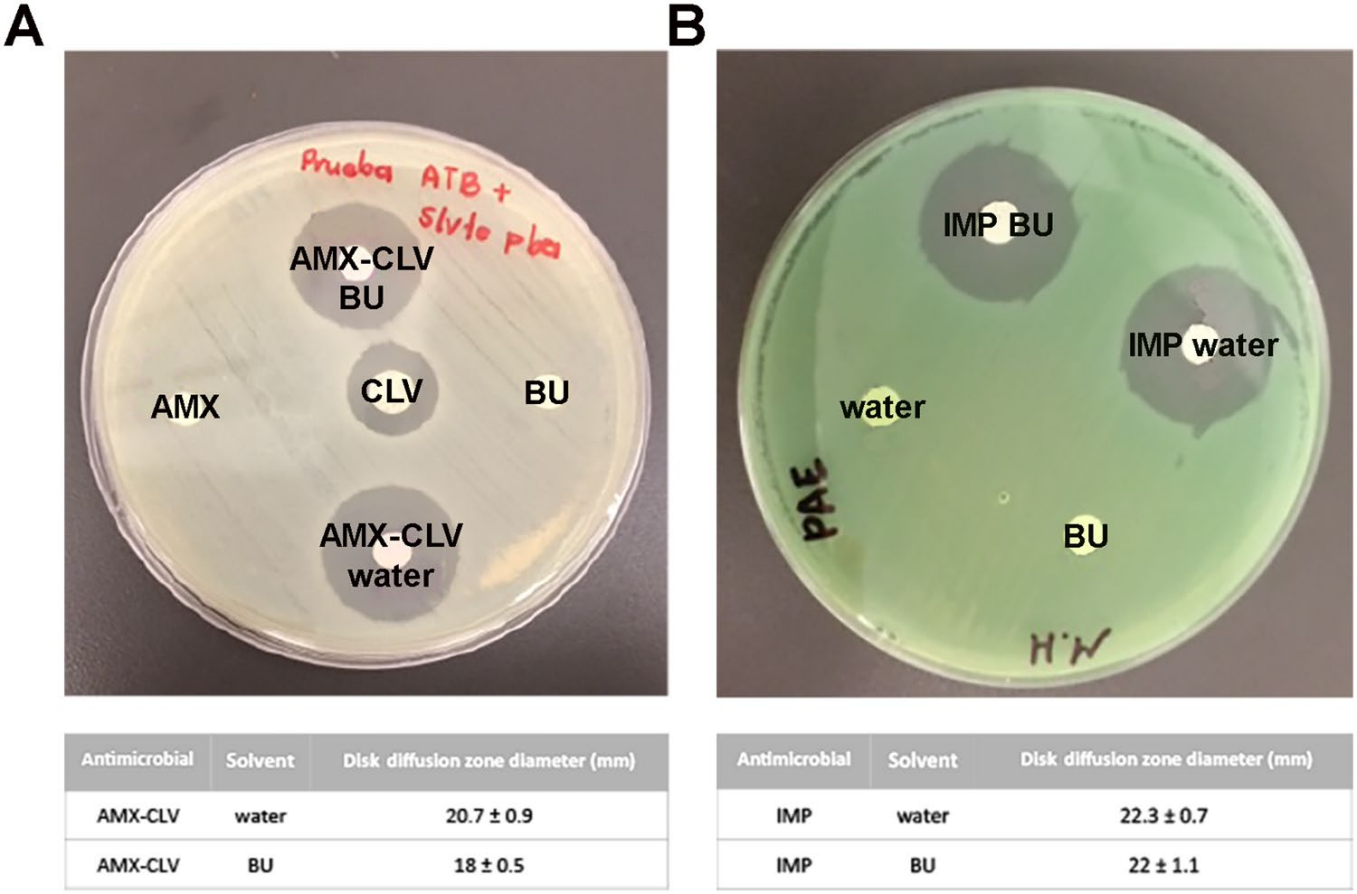
Representative inhibition zone assay on *Escherichia coli* ATCC-35218 cultures using membrane disks loaded with clavulanic acid together with amoxicillin in water solution (AMX-CLV water); amoxicillin clavulanic acid in BU solutions (AMX-CLV BU) and clavulanic acid (CLV), amoxicillin (AMX) and BU membrane disks as controls (**A**). Representative inhibition zone assay on *Pseudomonas aeruginosa* ATCC-27853 cultures using membrane disks loaded with imipenem in water solution (IMP water); imipenem in BU solutions (IMP BU) and BU membrane disks as control. Results are expressed as median ± SEM of three experimental replicates.

Figure 8 shows two representative plate images with CLV (Fig. 8A) and IMP (Fig. 8B) inhibition zones results. Note that to strictly follow CLSI specifications, CLV was evaluated together with AMX. The inhibition zone diameters of both CLV and IMP dissolved in water or in BU are very similar. Thus, confirming those antimicrobial activities were not affected by dissolving the antibiotics in BU.

Considering that the DD test needs the diffusion of the drugs loaded onto the cellulose membranes in order to generate the inhibition zone, it can be concluded that both CLV and IMP antibiotic molecules are available, meaning that they are not restricted to exert their action or to diffuse when dissolved in BU.

## Conclusion

We formulated and characterized a natural nonconventional solvent (BU) that increases the stability of two of the most unstable therapeutic β-lactam molecules: CLV and IMP. The obtained solvent was able to stabilize the drug molecules in solution without affecting their antimicrobial activity for seven days in contrast to use of water. These results suggest that BU could be an interesting alternative solvent for clinical preparations. However, additional experiments are needed to confirm the biological compatibility of BU formulations.
